# Multi-method joint monitoring study on strata behavior in shallow seam mining under bedrock gully

**DOI:** 10.1038/s41598-023-41877-w

**Published:** 2023-09-15

**Authors:** Jing Chai, Zhe Ma, Dingding Zhang, Jianfeng Yang, Zhiming Huang, Fengqi Qiu

**Affiliations:** 1 School of Energy, Xi’an University of Science and Technology, Xi’an, 710054 China; 2 Western Key Laboratory of Mine Mining and Disaster Prevention, Ministry of Education, Xi’an University of Science and Technology, Xi’an, 710054 China

**Keywords:** Geomorphology, Meteoritics, Mineralogy

## Abstract

The Baijigou Coal Mine in Helan mountain mining area is alpine gully landform, and the double key stratums are cut by the gully. A two-dimensional physical similarity model under this condition was established in the laboratory. The strain field, deformation field and pressure field of the model are jointly and accurately monitored by BOTDA, DIC and pressure sensors. The rock behavior in shallow coal seam mining under bedrock gullies are studied. In the mining stage under the gully, the deformation of overburden is intense because overburden is thin, and the surface is easy to form a depression basin. In the mining stage far away from the gully, the deformed rock mass lacks horizontal support in the process of deflecting to the lower goaf, and the movement rock is in a multilateral block. The multilateral block rock periodically deflects and rotates, resulting in the periodic deformation and break of the inferior key stratum. At the mining stage of away from the gully, nine times of roof weighting occurred. And there are large and small periodic weighting phenomenon with the average roof pressure concentration coefficient is 1.37. The distance between the peak point of advance abutment pressure and the coal wall is 6–18 cm, and the influence distance decreases with the advancing of the working face. The inferior key stratum has a significant impact on the weighting process and the weighting strength. In the mining stage under the mountaintop, large downward inclined tension crack is prone to produce in the slope on the side of the stope line, and the roof weighting is the most severe. In the mining stage close to the gully, the overburden falls in layers from bottom to top, and the overburden at the slope toe is prone to slip. At the mining stage under the mountaintop and close to the gully, five times of roof weighting occurred. And the roof weighting concentration coefficient is 1.46 on average. The distance between the peak point of advance abutment pressure and the coal wall is always kept at 6–16 cm, and the influence distance is 33–41 cm. The study can provide a reference for mining design and ensures safe and efficient mining in this condition.

## Introduction

There are a large number of shallow-buried coal seams with an average burial depth of less than 150 m in western China. And a large number of gullies on the surface, mainly distributed in Shendong mining area at the border of Shaanxi and Mongolia and Helan mountain mining area in northwest Ningxia. In Shendong mining area, the sand and soil layer of overburden is thick and the bedrock is thin. There is only a single key stratum in the overburden, and mainly mined by underground mining. A large number of mining practices show that under the sandy gully landform, when mining shallow coal seams, the pressure of the working face is stronger than that of the common buried face. And there are often dynamic load disasters such as the destruction of the working face supports, large area roof fall and coal pillar collapse, which seriously threaten the safe and efficient mining of the mine^1–3^. Helan mountain mining area in northern Ningxia used to adopt open-pit mining. Because it is located in Helan mountain natural ecological protection area, it has gradually changed to underground mining in recent years. Different from Shendong mining area, there are double key strata of the overburden. The surface bedrock is exposed without loose layer covering. The sensitivity of bedrock gullies to the influence of rock behavior is rarely involved in the existing research. Therefore, it is urgent to pay attention to the strata behavior in shallow coal mining under bedrock gullies.

At present, many scholars have studied the strata behavior when mining shallow coal seam under sandy gully. Savage et al. studied the stress distribution of isotropic rock mass under different topographical conditions, and concluded that stress concentration is easy to form at the bottom of the gully and at the foot of the slope^4–9^. Yang studied the fracture law of coal and rock under different pressures^10,11^. Zhang pointed out that the greater the trench depth, the closer the main key stratum is to the coal seam, the greater the gully slope angle, and the smaller the angle between the projection line of the gully slope strike and the advancing direction of the working face, the more vulnerable the working face is to the impact of dynamic load^12^. Wang divided the gully into sandy soil type and bedrock type, and he believed that the larger the slope angle, the smaller the bedrock thickness, and the more likely the multilateral block hinge structure of the mining slope to slide^13–15^. Wang believed that the rotation and breaking of key stratum beneath gully bottom under nonuniform load is the fundamental cause of strong dynamic strata behaviors^16^. Ju et al. studied the temporal and spatial distribution and formation mechanism of surface mining cracks in shallow coal seam mining in the gully area, and believed that the impact of topography and geomorphology on the roof caving law of coal seam is significant in the gully area^17–23^. Wang et al. studied the development law of water conducting fracture zone in shallow coal seam under gully topography^24^. Bai et al. believed that under the mining action of shallow coal seams in the sandy gully area, the overburden formed a double-layered block distribution structure with a length of large at the top and small at the bottom^25–29^.

The research methods for the deformation and movement of overburden in coal mining mainly include theoretical calculation^30,31^, physical simulation of similar materials^32–35^, numerical simulation experiment^36,37^ and field measurement^38–40^, etc. The deformation of overburden caused by mining is a typical grey structure problem^41^. Under this background, the similar material model test is highly concerned by many scholars because it can visually and dynamically reflect the development process of overburden strata. Its significance is to enable us to conduct data monitoring and law research on the problems through advanced monitoring technology and combining with real experimental phenomena. Then the conclusions and mature monitoring technology will be further applied to the field. In recent years, with the rapid development of various optical sensing technologies, optical fiber sensing technology and digital image correlation method (DIC) have been applied to physical similarity model experiments. The optical fiber is usually embedded in the model to make it synchronously deform with model. The deformation process and characteristics of the model are deduced by measuring the strain of the optical fiber. And the high-precision and distributed monitoring of the internal deformation process of the overburden is realized^42–46^. DIC is mainly used for high-precision measurement of model surface strain field and displacement field^47,48^. It can be seen that the development of optical sensing technology has greatly promoted the progress of physical model experiments.

According to the surface morphology and geological conditions of Baijigou Coal Mine in Helan mountain mining area, a model size of 272 cm × 133 cm × 20 cm (length × high × wide) was made. And then, the strain field, deformation field and pressure field of the model are jointly monitored by Brillouin optical time-domain analysis system (BOTDA), DIC and pressure sensors. The strata behaviors are studied when mining shallow coal seam under bedrock gully with both key strata cut by the gully. The study can provide a reference for mining design and ensures safe and efficient mining in this condition.

## Background

### The principle of BOTDA

**Figure 1:**
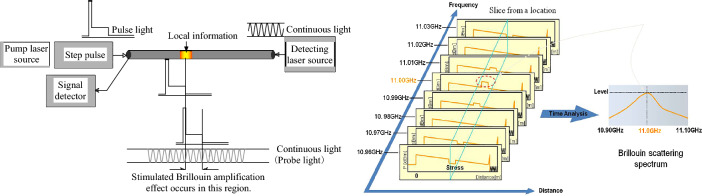
Working principle of Brillouin optical time domain analysis.

The frequency of Brillouin scattering light can be affected by the temperature and strain of the fiber when the light is transmitted in the optical fiber. Therefore, the change value of temperature and strain can be obtained by detecting the change of Brillouin scattering light frequency. This test adopts the PPP-BOTDA optical fiber sensing technology based on stimulated Brillouin scattering. Inject pulse light at one end of the fiber and continuous light at the other end. If the frequency difference between two beams is equal to the Brillouin frequency shift at a certain position of the optical fiber, energy transfer occurs between the two beams. By detecting the frequency of the continuous light after energy exchange with the pulse light, the frequency variation of stimulated Brillouin scattering light at each position of the fiber can be determined, and then the strain and temperature values can be calculated. The variation of Brillouin frequency shift is linear with strain and temperature, as shown in Formula (1). The measurement principle and system architecture of BOTDA are shown in Fig. 1.

The relationship between Brillouin frequency drift and strain and temperature variation is as follows:

1 $$ V_{{\text{\rm B}}} (\varepsilon ,\;{\text{\rm T}}) = V_{{\text{\rm B}}} (0) + \frac{{dV_{{\text{\rm B}}} (\varepsilon )}}{d\varepsilon }\varepsilon + \frac{{dV_{{\text{\rm B}}} ({\text{\rm T}})}}{{d{\text{\rm T}}}}({\text{\rm T}} - {\text{\rm T}}_{0} ) $$

where *V*_*B*_ (*ε*, *T*) is the drift of fiber Brillouin frequency shift with a certain strain or temperature; and *V*_*B*_ (*0*) is the drift of fiber Brillouin frequency shift without strain and temperature; ε is the axial strain of sensing fiber; *T* *−* *T*_*0*_ is the change of temperature; *dV*_*B*_ (*ε*)*/dε* and *dV*_*B*_ (*T*)/*dT* are strain and temperature sensitivity coefficients respectively.

### The principle of DIC

**Figure 2:**
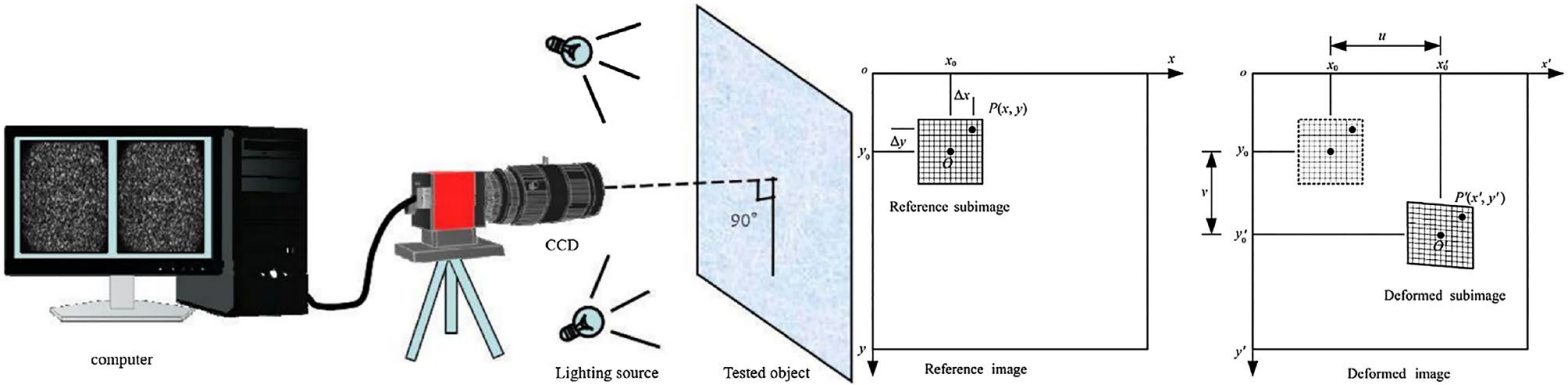
The system and working principle of DIC. Schematics of the reference and deformed subsets.

As shown in Fig. 2, DIC is a non-contact modern optical measurement experimental technology, which is composed of CCD camera, lighting source, image acquisition card and computer. The system has many advantages, such as simple light path, good environmental adaptability, wide measuring range and high degree of automation. The basic principle is to obtain the displacement vector of the pixel by tracking (or matching) the position of the same pixel in the two speckle images before and after the object surface deformation, which can realize the full field strain and displacement monitoring of the model surface.

## Introduction of the experiment

### Physical model design

**Figure 3:**
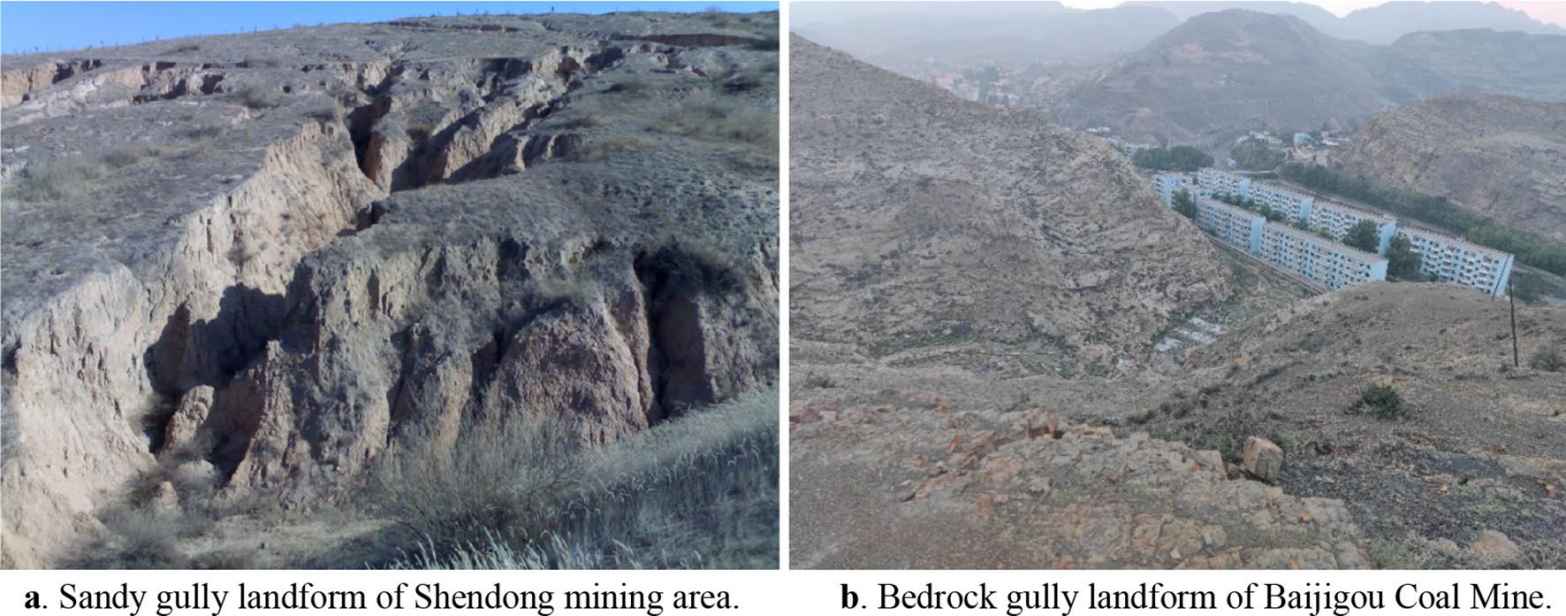
Two main gully landforms.

Two different types of gully landforms are shown in Fig. 3, of which Fig. 3a is sandy gully landform of Shendong mining area and Fig. 3b is bedrock gully landform of Baijigou Coal Mine. The first layer of the working face numbered 010204 is currently being mined in Baijigou Coal Mine, with a strike length of 350 m, a dip width of 195 m, and a mining height of 3 m. The roof of the coal seam is 60 m away from the surface valley and 180 m away from the mountaintop. The extension direction of the valley is perpendicular to the strike of the working face, and the inclination angle of the mountain slope is about 45°.

**Figure 4:**
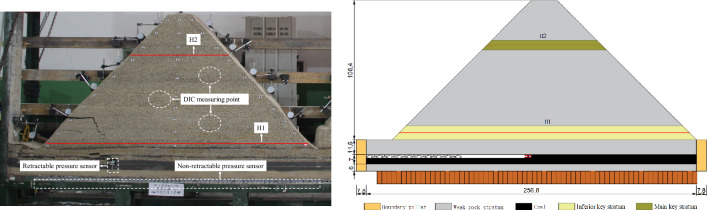
Model size and sensors layout (unit: cm).

The physical similarity model is established based on the terrain characteristics of Baijigou coal mine in Helan mountain mining area. The rock stratum distribution is obtained by sampling in the vertical borehole at the site, and the strength parameters of each rock stratum are obtained through the mechanical property test. River sand, gypsum and white powder are used as similar materials, and the ratio of similar materials for each rock stratum is determined through orthogonal experiments. The overburden lithology and thickness are shown in Table 1. The coarse-grained sandstone of the 10th layer is the lower key stratum and the 30th layer is the upper key stratum. The two key stratums are cut by gullies and exposed to the surface. The geometric similarity ratio of the model is 1:200, the unit weight similarity ratio is 1:1.56, the stress similarity constant is 312, and the time similarity constant is 14.14. The model is shown in Fig. 4. The thickness of coal seam floor is 6 cm and the coal seam is 7 cm. The layered mining is adopted and the first layer is 3 cm. The distance between the roof of the coal seam and the gully and the top of the mountain is 11.6 cm and 120 cm respectively. The slope of the mountain is 45°. The coal seam is mined from left to right. The width of boundary coal pillar is 7.8 cm, the width of open-off cut is 10 cm. The advance length of the first layer is 256 cm, and each excavation is 3 cm, with a total of 82 times.

**Table 1:** Physical parameters of overburden.

Number	Lithology	Thickness (cm)	Cumulative thickness (cm)	Number	Lithology	Thickness (cm)	Cumulative thickness (cm)
38	Coarse grain sandstone	4.1	133.4	19	Fine sandstone	2.7	65
37	Medium grain sandstone	2	129.3	18	Coarse grain sandstone	1	62.3
36	Coarse grain sandstone	3.8	127.3	17	Medium grain sandstone	1.8	61.3
35	Medium grain sandstone	6.8	123.5	16	Fine sandstone	4.5	59.5
34	Fine sandstone	1.7	116.7	15	Medium grain sandstone	1.2	55
33	Medium grain sandstone	2.8	115	14	Coarse grain sandstone	7.3	53.8
32	Coarse grain sandstone	4.9	112.2	13	Fine sandstone	7	46.5
31	Medium grain sandstone	4.9	107.3	12	Medium grain sandstone	2	39.5
30	Coarse grain sandstone	7.5	102.4	11	Fine sandstone	2	37.5
29	Medium grain sandstone	3	94.9	10	Coarse grain sandstone	10.8	35.5
28	Coarse grain sandstone	4.8	91.9	9	Fine sandstone	1	24.7
27	Medium grain sandstone	2.2	87.1	8	2–1 coal	1.3	23.7
26	Coarse grain sandstone	5.2	84.9	7	Siltstone	1.6	22.4
25	Medium grain sandstone	1	79.7	6	Coarse grain sandstone	4.1	20.8
24	Coarse grain sandstone	3.9	78.7	5	2–2 coal	1.1	16.7
23	Medium grain sandstone	1.9	74.8	4	Siltstone	1.3	15.6
22	Coarse grain sandstone	4.9	72.9	3	Coarse grain sandstone	1.3	14.3
21	Medium grain sandstone	2	68	2	2–3 coal	7	13
20	Coarse grain sandstone	1	66	1	Coarse grain sandstone	6	6

### Test system

The sensor deployment scheme is shown in Fig. 4. Distributed optical fibers are embedded inside the model to obtain deformation characteristics of key strata inside the model. DIC is used to obtain the full field deformation features of the model surface. The pressure sensor obtains the stress changes of the roof and floor after coal seam mining.

Two optical fibers are horizontally arranged on the inferior and main key stratums, and are named H_1_ and H_2_ respectively. The temperature in the laboratory is different from that in the model. Since the sensing optical fiber is also sensitive to temperature, another optical fiber is buried in the boundary coal pillar, where the model will not be disturbed by mining for temperature compensation. The distributed optical fiber sensing test system is mainly composed of NBX-6055 optical fiber demodulator, computer and sensing optical fiber. Among them, NBX-6055 optical fiber demodulator and computer are used to collect and demodulate signals. The sensing optical fiber is single-mode optical fiber with the diameter of 2 mm. The parameter settings of the optical fiber test system in this test are shown in Table 2.

**Table 2:** Major technical performance indices of NBX-6055 during overburden monitoring.

Measurement distance (m)	Sampling interval (cm)	Frequency interval (MHz)	Averaging count	Strain range (%)	Pulse width (ns)	Spatial resolution (cm)	Accuracy (με)	Output probe power (dBm)	Output pump power (dBm)
50	1	5	2^16^	− 3 to 4	0.5	5	7	0 to 3	27 to 30

The ARAMIS 3D digital speckle equipment from Dome, Germany, was selected for the test. The system mainly includes Canon EOS700D camera with image resolution of 18 million pixels, two sets of white LED light sources, one computer and 3D-DIC analysis software. In order to reduce the influence of artificial speckle on the physical properties of similar model test materials and ensure that the speckle pattern is consistent with the deformation of the measured object surface, a circular speckle is drawn on the model surface by using a brush and ink. The size of speckle is relatively uniform, with a diameter of about 3 mm. The location of speckle on the model surface is random, and the speckle density is about 40%.

After each excavation, 1 set of retractable pressure sensing support is arranged behind the coal wall. By adjusting the height to make it fully contact with the roof and floor, the roof is supported while monitoring the real-time load of the working face. Bracket size is 20 cm × 3 cm (length × width), surface area is 60 cm^2^. In this test, the initial support force is 0.17 Mpa, and the measure scope is 0.27 MPa. The calibrated CL-YB-114 type non-retractable pressure sensor is embedded under the coal seam floor to monitor the abutment pressure in real time. The sensors are arranged horizontally along the model, with a total of 66 sensors, numbered 1–66 # from left to right. The model of data acquisition instrument is AD-64/MV.

## Results and discussion

### Characteristics of overburden movement

The model is excavated from left to right along the strike. As the model is excavated, the working face successively goes through under gully, far away from the gully, under the mountaintop and close to the gully. In this test, the overburden surface strain nephogram obtained by DIC is used to analyze the deformation and movement characteristics at different mining stages.

**Figure 5:**
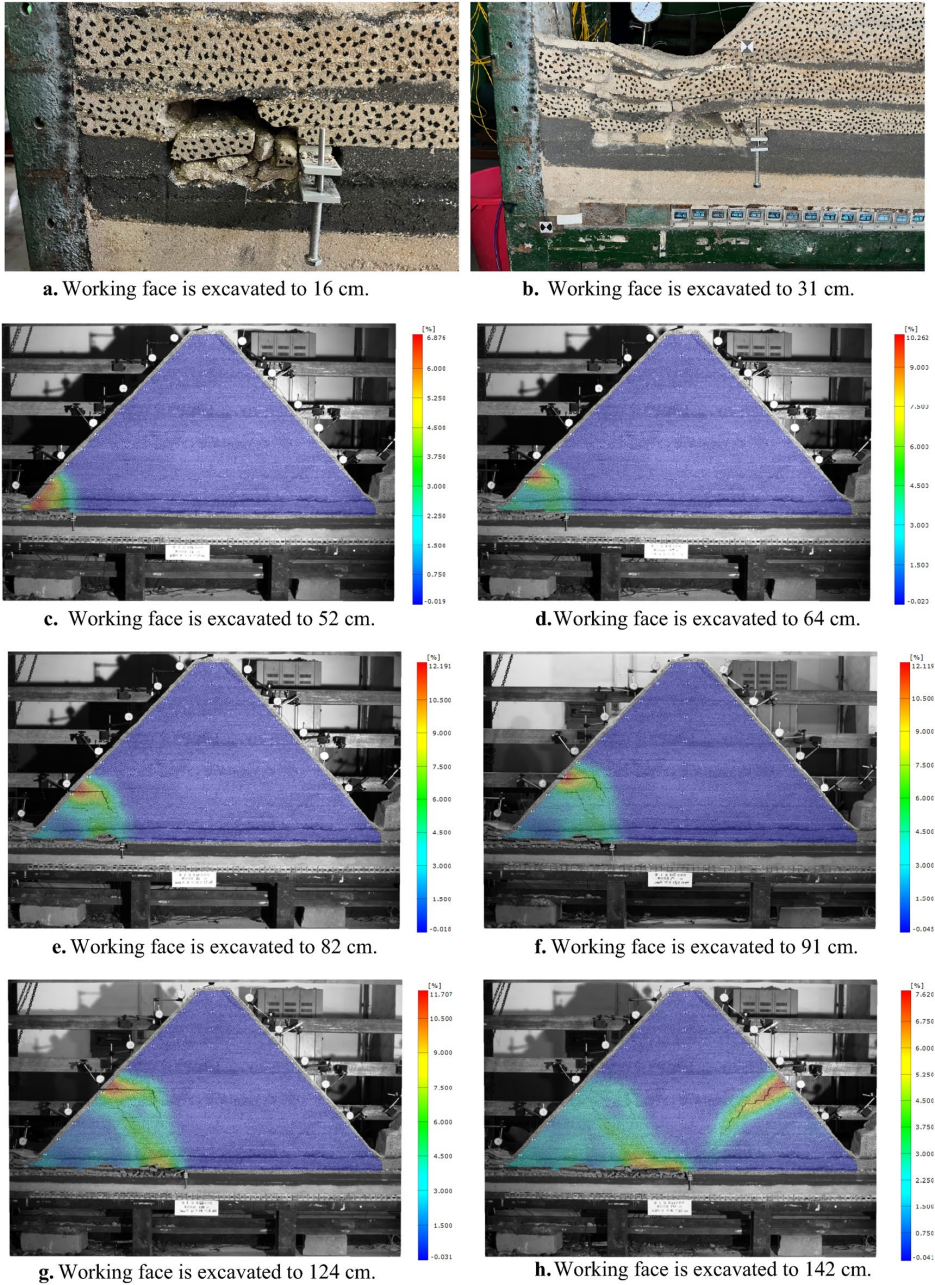

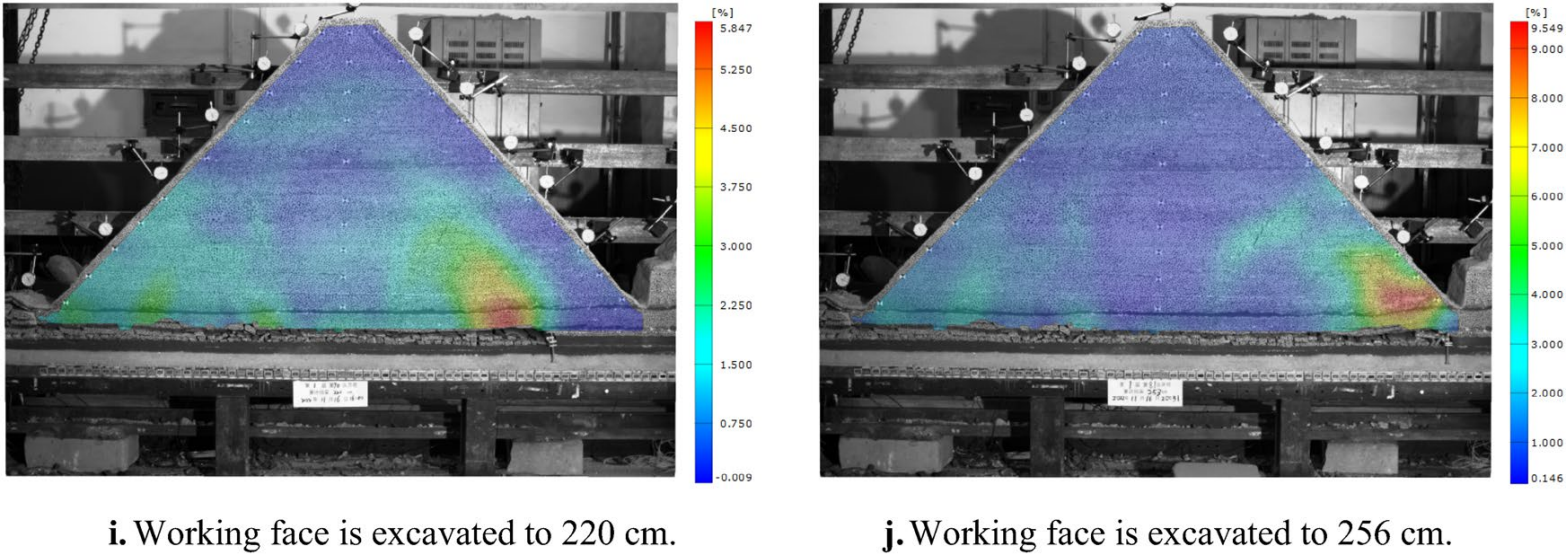
Strain nephogram of overburden at different mining stages.

Figure 5 shows the overburden strain nephogram at different mining stages. When mining under the gully, the movement of overburden is intense, and the camera cannot identify the survey points. From the experimental process, when the working face is advanced to 16 cm, the direct roof collapsed for the first time, and the irregular rock blocks fell into the goaf in disorder. When the working face is advanced to 31 cm, the main roof is broken for the first time. The broken rock mass is squeezed by the undeformed rock mass on both sides, forming a relatively stable bearing structure. The rock strata over the main roof is weak, and they bend, sink and break along with the main roof. The ground surface at the front and rear boundary of the goaf forms downward stretching cracks respectively, which are connected with the goaf. The rock masses cut by fractures slip and subside towards the lower goaf, and the surface forms a basin.

The deformed rock masses of the mountain lack lateral displacement constraints during horizontal movement, and the broken rock masses can’t form a bearing structure by articulating with each other. Therefore, the movement and fracture characteristics of the overburden have changed significantly in the mining stage of far away from the gully. When the working face is advanced to 52 cm, 64 cm, 82 cm, 91 cm and 124 cm respectively, five horizontal separations are generated at the junction of the soft and hard rock layers at the height of 34 cm, 37 cm, 48 cm, 56 cm and 66 cm on the left slope. The rock masses below the separation layer deflects to the lower left goaf, which incline crack occurs at the right end of the separation and propagates downward. Therefore, a multilateral block rock structure is formed below the separation. The main key stratum is thick and hard, which hinders the separation and crack develop to the higher position. In the mining stage under the mountaintop, when the working face is advanced to 142 cm, the whole overburden deflected to the lower left, resulting in inclined tension cracks under the main key stratum of the right slope of the mountain, and continues to expand downward.

In the mining stage close to the gully, with the advance of the working face, the mining overburden continuously deflected to the newly formed goaf at the lower right. At this time, the undeformed overburden on the right limit the horizontal movement of the mined overburden on the left. Therefore, the mining overburden continuously bends, breaks and collapses to the lower goaf layer by layer from bottom to top. When the excavation of the working face is close to the right slope toe, the overburden above the slope toe is deformed. Since the gully on the right side of the mountain can’t provide displacement constraints, the overburden at the slope toe slipped.

### Deformation characteristics of key strata

The key stratum is the thick and hard rock stratum of the overburden, which has good bearing capacity. When the key stratum is intact, the rock stratum above it will not settle. When the key stratum is broken, the weak rock stratum above it and the key stratum subsides synchronously. The key stratum is divided into main key stratum and sub key stratum. There can be multiple key stratums, but there is only one main key stratum. If the surface subsidence occurs after a key stratum is broken, then the rock stratum is the main key stratum.

The optical fiber in the model is closely contact with the rock strata, and they deform synchronously. Therefore, the stress state of the optical fiber is the stress state of the measured overburden. A large number of test results prove that when the fiber is compressed along the radial direction, the Brillouin frequency shift value is negative, and when the fiber is pulled along the radial direction, the Brillouin frequency shift value is positive^49,50^. And the frequency shift value is linear with the strain. Therefore, the strain obtained through the optical fiber frequency value can further deduce the stress state and deformation collapse characteristics of overburden. In this test, the deformation process and characteristics of inferior key stratum and main key stratum are characterized by the strain changes of H_1_ and H_2_ optical fibers.

**Figure 6:**
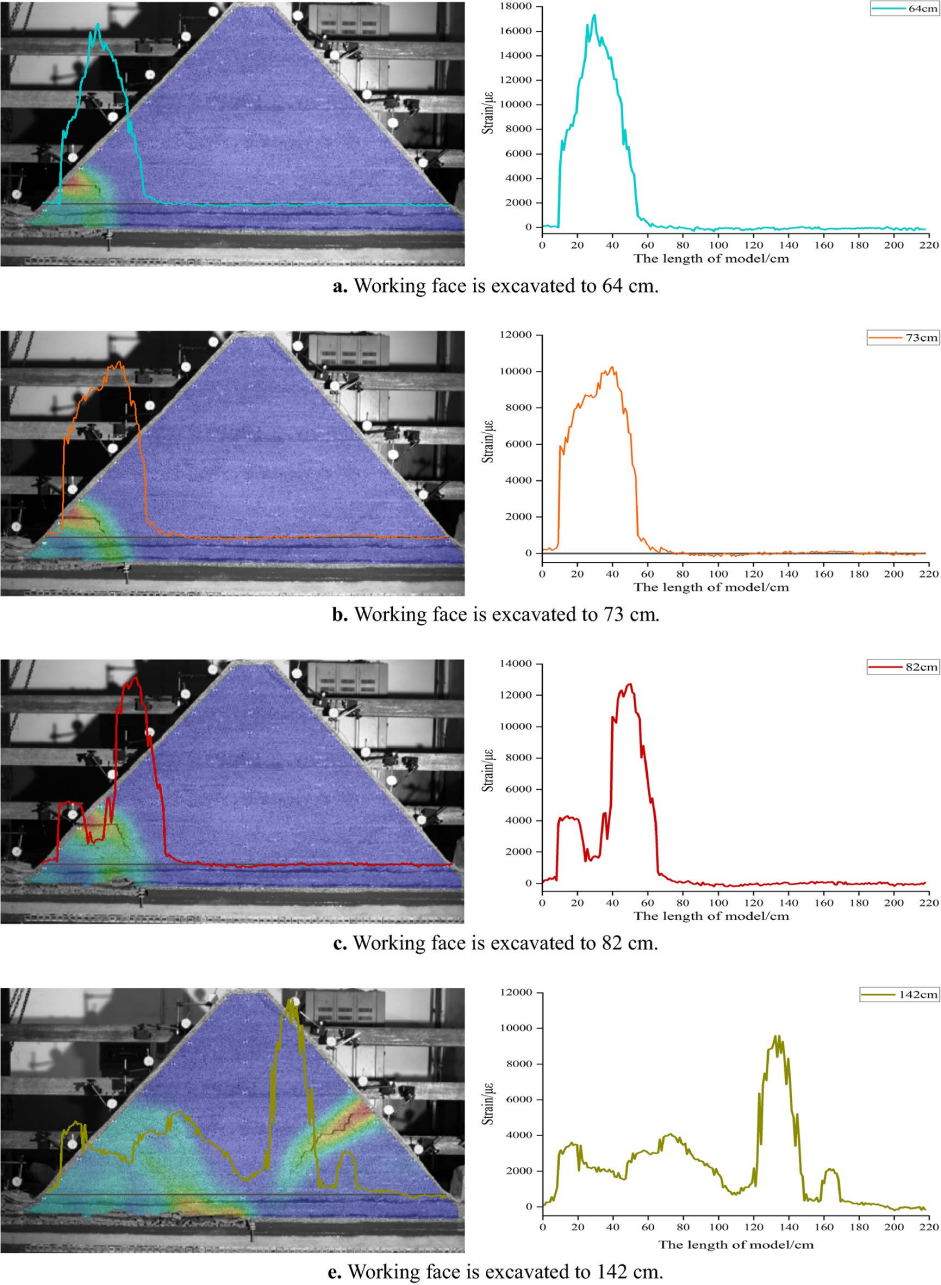

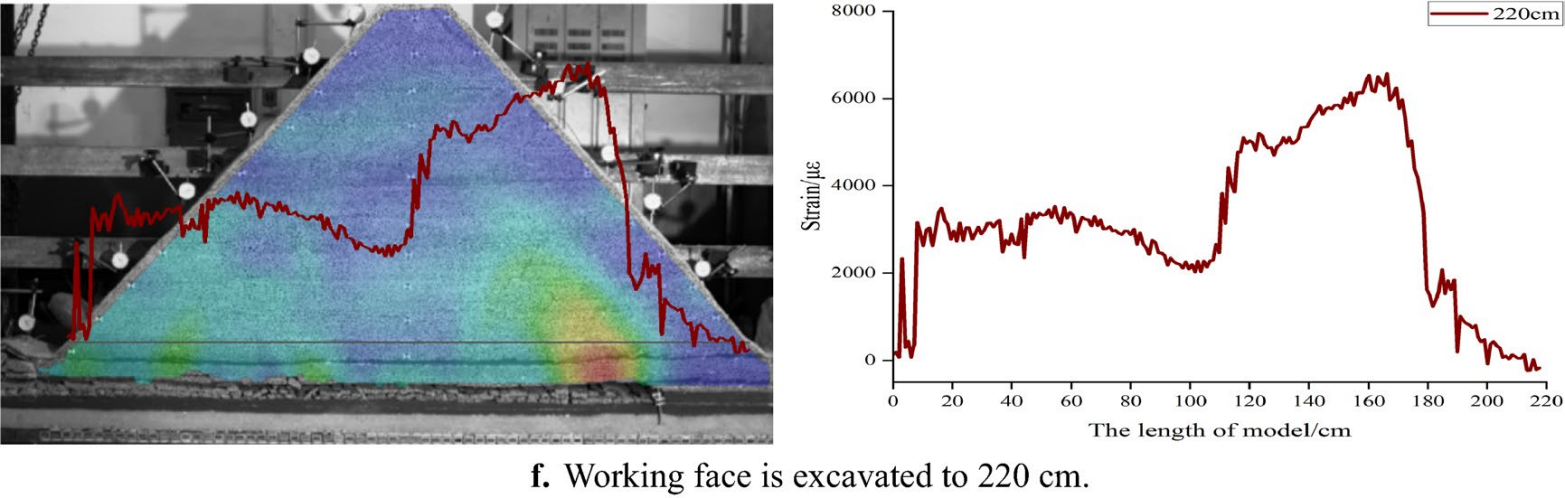
Corresponding position relationship between strain curve of H_1_ optical fiber and lower key stratum.

**Figure 7:**
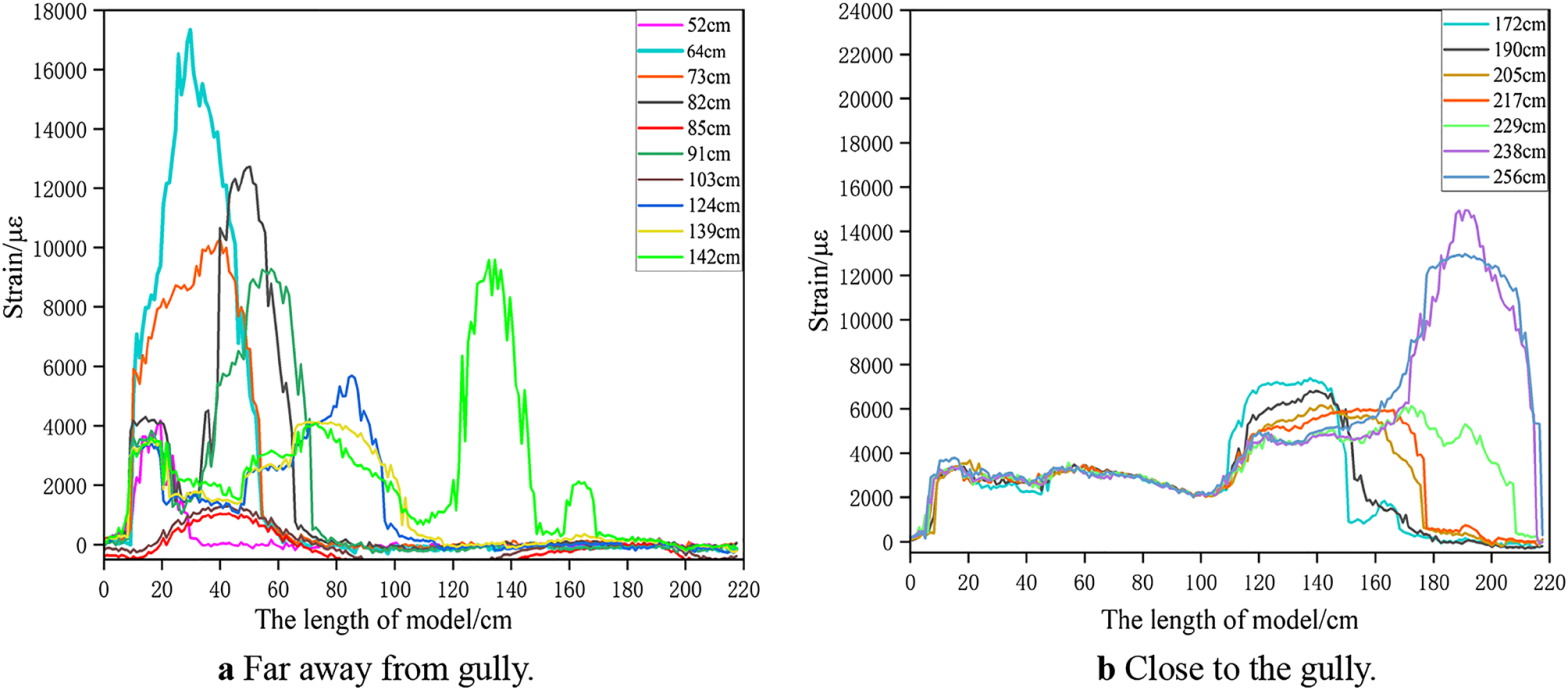
Strain curve of optical fiber H_1_ at different mining stage.

Figure 6 shows the corresponding position relationship between H_1_ fiber strain curve and inferior key stratum. Figure 7 shows the change of H_1_ optical fiber strain. On the whole, the strain curve shows a single peak before the working face advances to 82 cm. When the working face is pushed to 82–142 cm, it shows a double-peak shape. And then, it shows multi-peak shape. The peak strain of the optical fiber corresponds to the deformation and fracture position of the inferior key stratum.

**Figure 8:**
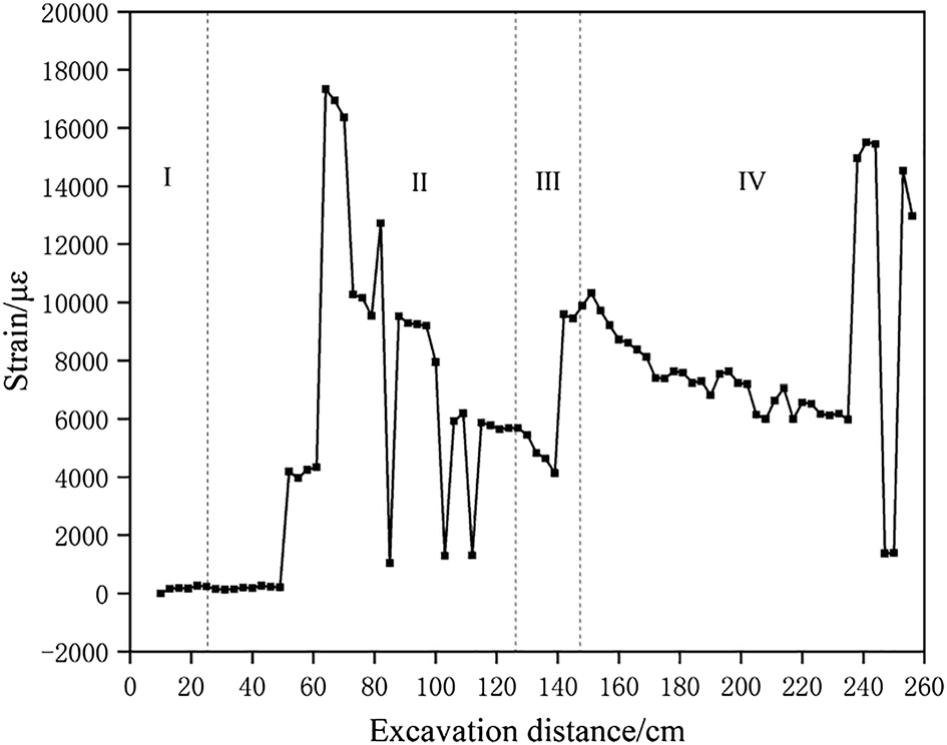
Strain peak change curve of H_1_ optical fiber.

Figure 8 shows the change of H_1_ optical fiber peak strain. The four mining stages correspond to under gully, far away from the gully, under the mountaintop and close to the gully respectively. In the mining stage far away from the gully, when the working face is advanced to 52 cm, the deflection of the multilateral block rock to the lower left goaf causes the deformation of the inferior key stratum, and the strain suddenly increased to 4189 με. When the working face is pushed to 64 cm, the strain increased rapidly to 17,336 με, indicating the inferior key stratum increased deformation. When the working face is advanced to 73 cm, the strain is significantly reduced to 10,189 με. It shows that the inferior key stratum broke and collapsed and the multilateral block rock rotated to the right goaf. A deformation and movement cycle of inferior key stratum ends. During the whole mining stage far away from the gully, the inferior key stratum has undergone four periodic deformation and break when the working face is pushed to 73 cm, 85 cm, 103 cm and 112 cm respectively.

In the mining stage under the mountaintop, when the working face is advanced to 142 cm, the strain increases significantly, indicating that the oblique tension crack on the right side of the mountain passed through the inferior key stratum and the inferior key stratum is broken. In the early and middle mining stage of close to the gully, the strain changes periodically and slightly, indicating that the inferior key stratum is periodically broken, but the settlement value is small. The break of inferior key stratum occurred when the working face is pushed to 172 cm, 190 cm, 205 cm, 217 cm and 229 cm respectively. The reason is no longer the deflection and rotation of the multilateral block rock, but the main roof breaks and collapses below it, which causes the inferior key stratum lose part of support. In the late mining stage of close to the gully, the inferior key stratum at the slope toe slipped, and the strain changes dramatically.

**Figure 9:**
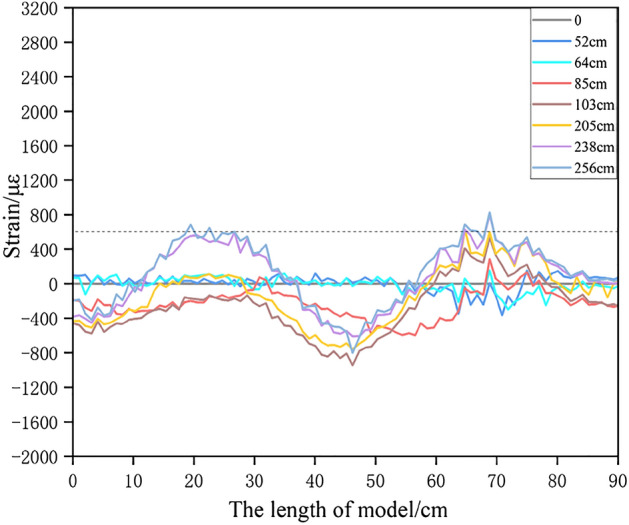
Strain curve of optical fiber H_2_ at different mining stage.

Figure 9 shows the H_2_ optical fiber strain at different mining stages. In the mining stage of far away from the gully, the main key stratum is compressed, and the stress rises slightly with the advancing of the working face. In the mining stage of close to the gully, both ends of the main key stratum are tensioned while the middle part is compressed. After the slope toe on the right side of the overburden slips, the maximum tensile strain at the left and right ends of the main key stratum exceeds 600 με. When microcracks occur in geotechnical materials, the optical fiber tensile strain is generally 600–1200 με^51,52^. Therefore, microcracks may occur in the main key stratum.

### Characteristics and mechanism analysis of rock pressure

The movement of the mining overburden will lead to the change of overburden stress field. The roof pressure concentration coefficient is defined to reflect the intensity of weighting. It is the ratio of the pressure after each coal seam excavation to the pressure before excavation. The expression is as shown in Formula (2):

2 $$ Y = \frac{{P_{N} }}{{P_{S} }} $$

where *Y* is the roof pressure concentration coefficient. *P*_*N*_ is the sensor pressure after each coal seam excavation; *P*_*S*_ Pressure before coal seam excavation.

**Figure 10:**
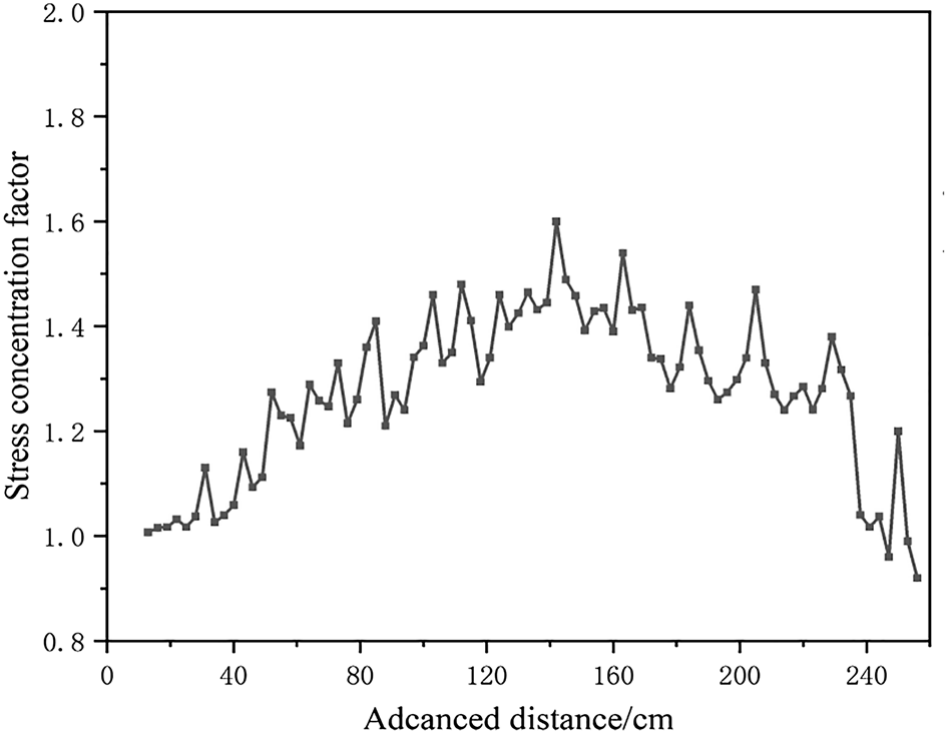
The change of working face support pressure.

Figure 10 shows the change of roof pressure on working face during the whole mining stage. At the mining stage of under the gully and away from the gully, eleven times of roof weighting occurred. When the working face is excavated to 31 cm, the first roof weighting occurred and the roof pressure concentration coefficient is 1.13. When the working face is advanced to 43 cm, the first periodic roof weighting occurred and the roof pressure concentration coefficient is 1.16. The collapsed rock block forms a stable bearing structure, and the inferior key stratum is not broken at this moment, so the roof weighting strength is small. In the mining stage far away from the gully, when the working face is pushed to 52 cm, 64 cm, 82 cm, 91 cm and 124 cm respectively, the multilateral block rock including inferior key stratum deflected to the lower left goaf, the roof weighting increased, and the weighting strength coefficients is 1.27, 1.29, 1.36, 1.27 and 1.46 respectively. When the working face is pushed to 73 cm, 85 cm, 103 cm and 112 cm respectively, the inferior key stratum is broken, and the multilateral block rock rotated to the lower right goaf. The roof weighting significantly increased, and the strength coefficient is 1.33, 1.41, 1.46 and 1.48 respectively. It can be seen that the roof has significant large and small periodic weighting phenomenon, and the inferior key stratum has a great impact on the weighting process and strength.

At the mining stage under the mountaintop and close to the gully, five times of roof weighting occurred. In the mining stage under the mountaintop, the overall overburden deflected to the lower left goaf when the tension crack appears on the right slope of the mountain. The roof pressure increased significantly, exceeding the maximum range of the support. In the early and middle mining stages of close to gully, the mining overburden on the left and right side of the crack rotated to the lower goaf, resulting in a significant increase in roof pressure. When the working face is pushed to 163 cm, 184 cm, 205 cm and 229 cm respectively, the strength coefficient factors is 1.54, 1.44, 1.47 and 1.38 respectively. In the late mining stage of close to the gully, the overburden at the foot of the slope on the right side of the mountain slipped and the roof pressure decreased significantly. And the strength coefficients factor is 1.04, 0.96 and 0.92 with the working face is pushed to 238 cm, 247 cm and 256 cm respectively.

The traditional working face has a layered failure of the overburden from bottom to top. After the main roof and key strata are broken, a stable articulated bearing structure can be formed, which limits the deformation and movement of the overlying rock layer. The movement of the overlying rock during mining is relatively gentle, and the strength of the roof pressure is small. During shallow seam mining under bedrock gully, the lack of overlying rock at the valley location results in a lack of horizontal displacement restrictions on the basic roof and key layers, making it difficult to form a stable articulated bearing structure by breaking rock blocks. Therefore, the movement of overlying strata during mining is more intense, the strength of roof pressure is greater, and dynamic loading of ore pressure may occur.

**Figure 11:**
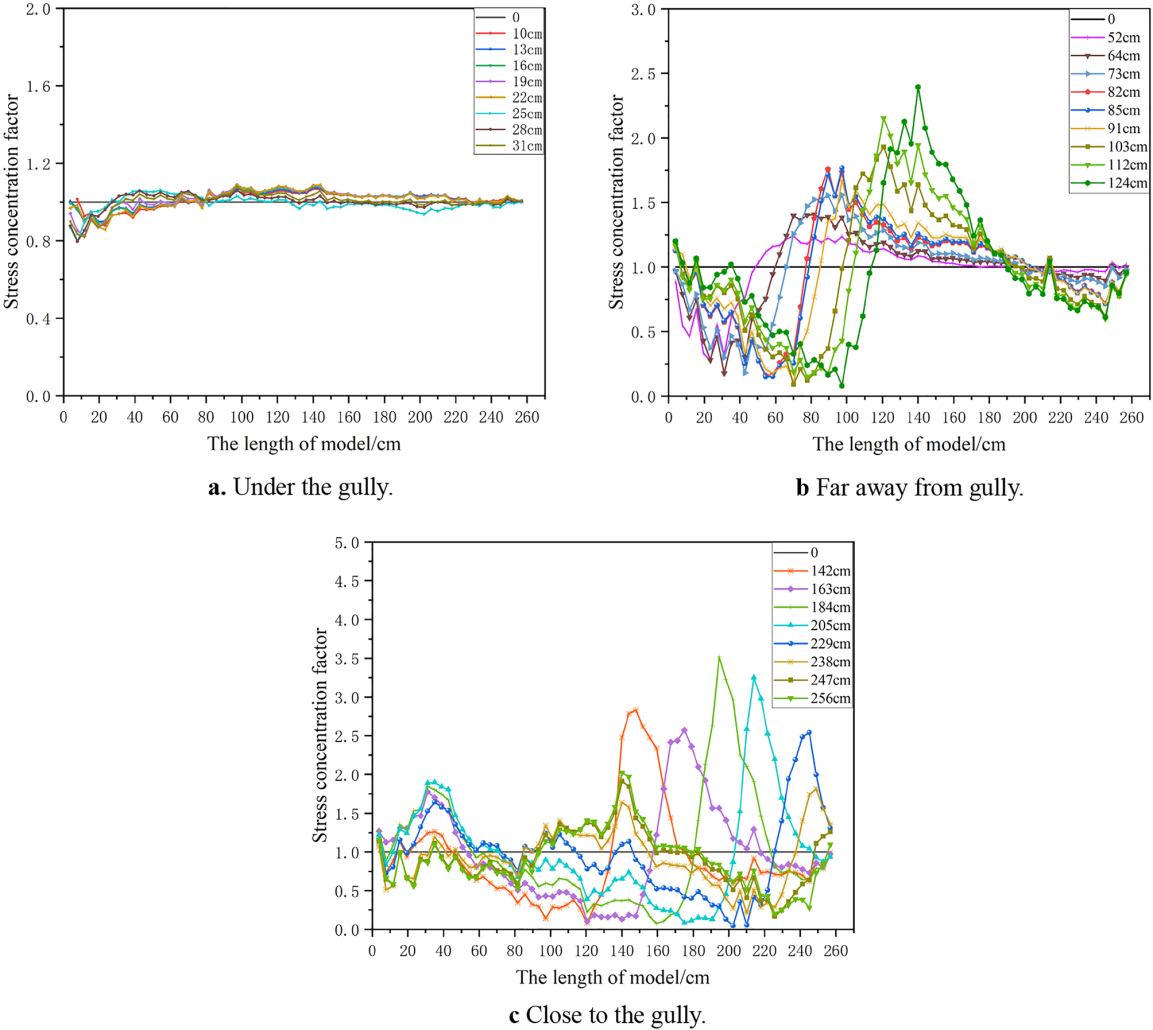
The pressure at the bottom of the model at different mining stages.

Figure 11 shows the change of coal seam floor pressure at different mining stages. After the coal seam is mined, the pressure in the goaf decreases, while the pressure in front of the coal wall increases, which is called the advance abutment pressure. The overburden under the gully is thin and the mining distance is small, so the pressure change is slightly. In the mining stage far away from the gully, the pressure of the goaf is significantly reduced and the advance abutment pressure is significantly increased compared with that before the coal seam is mined. The transition point of pressure reduction and increase is always behind the support. It is significantly different from the traditional working face that the influence range of the advance abutment pressure doesn’t move forward with the advancing of the working face, and is always about 60 cm away from the boundary coal pillar at the side of the stope line. The distance between the peak point of pressure and the coal wall is always kept at 6–18 cm, so the influence distance of advance abutment pressure decreased with the advance. And then, the pressure in the goaf gradually recovers, and the advance abutment pressure continues to rise. The proof roof pressure concentration coefficient is 1.26 when the working face is advanced to 52 cm, and rose to 2.39 when advanced to 124 cm. In the mining stage close to the gully, the overburden rotated downward to the lower right, and the influence range of the advance abutment pressure moved forward with the advance. The influence distance of advance abutment pressure is 33–41 cm, and the distance between the peak point and the coal wall is 6–16 cm. The advance abutment pressure reaches the maximum when advanced to 172 cm, and the roof pressure concentration coefficient is 3.52, and restored to 1.81 when the working face is pushed to 238 cm. After the working face is pushed to 238 cm, the right slope toe of the mountain slipped, and the pressure at the corresponding position was significantly lower than that before the coal seam was mined.

## Conclusion

1. When mining under the gully, the overburden deformation is severe, and the fissures are easy to develop to the surface. In the mining stage far away from the gully, the mining overburden lacks horizontal support during the process of deflecting to the lower goaf, and the movement overburden is in a multilateral block. The multilateral block rock deflects to the goaf at the side of the open-off cut, and then rotates to the goaf at the side of the stope line after the inferior key stratum is broken. In the mining stage under mountaintop, inclined tension cracks are easy to occur on the side slope of the stope line. When mining close to gully to the slope toe, the overburden is prone to slip.
2. The causes of deformation and fracture of inferior key stratum are different at different stages. At the mining stage of away from the gully, the multilateral block deflects and rotates periodically, resulting in the deformation and break of the inferior key stratum. In the mining stage of close to the gully, the main roof is broken, the inferior key stratum loses its support and breaks.
3. During the whole mining stage, sixteen times of roof weighting occurred. In the mining stage far away from the gully, the inferior key stratum causes the roof to have significant large and small periodic weighting phenomenon. When the multilateral block rock deflects towards the goaf at the side of the open-off cut, the weighting strength is relatively small. When the multilateral block rock rotates to the goaf at the side of the stope line, the weighting strength is relatively large. The roof weighting strength at the mining stage close to the gully is greater than that at the mining stage far away from the gully.
4. The distance between the peak point of advance abutment pressure and the coal wall is 6–18 cm. The peak value is reached peak when the working face is advanced to 172 cm, and the roof pressure concentration coefficient is 3.52. In the mining stage far away from the gully, the influence distance of the advance abutment pressure decreases with the advance of the working face. In the mining stage close to the gully, the influence distance of advance abutment pressure is 33–41 cm.

## Data Availability

The datasets generated during the current study are not publicly available. Because these data are part of our research project, which is currently under way. We must wait until this research project is completed before we can make all the data public. But the data in this paper are available from the corresponding author on reasonable request.
